# Recent Advances in Intermediate Filaments—Volume 1

**DOI:** 10.3390/ijms23105308

**Published:** 2022-05-10

**Authors:** Angela Saez, Jose M. Gonzalez-Granado

**Affiliations:** 1 LamImSys Lab, Instituto de Investigación Sanitaria Hospital 12 de Octubre (imas12), 28041 Madrid, Spain; 2 Facultad de Ciencias Experimentales, Universidad Francisco de Vitoria (UFV), 28223 Pozuelo de Alarcón, Spain; 3 Departamento de Fisiología, Facultad de Medicina, Universidad Autónoma de Madrid (UAM), 28029 Madrid, Spain; 4 Centro Nacional de Investigaciones Cardiovasculares (CNIC), 28029 Madrid, Spain; 5 Centro de Investigación Biomédica en Red de Enfermedades Cardiovasculares (CIBERCV), 28029 Madrid, Spain

We would like to make readers of the second edition of the Special Issue from the *International Journal of Molecular Sciences* on the Recent Advances in Intermediate Filaments aware of the content of the first edition on this same topic.

The cytoskeleton is composed of three different protein networks: microfilaments with a diameter of 6 nm that formed from polymers of actin, microtubules with a diameter of 25 nm that consist of tubulins, and intermediate filaments with a diameter of 10 nm that form from intermediate filament proteins (IFs).

IFs are coded by over 70 differentially expressed genes in a cell- and tissue-specific manner. IF expression is also affected by the developmental stage of cells and tissues and by a variety of disease conditions. IFs are classified into six subgroups, namely type I to type VI, based on similarities in the amino acid sequence and molecular structure. In detail, acidic keratins are type I IFs; basic keratins are type II IFs; desmin, GFAP, peripherin, vimentin, and syncoilin are type III IFs; nestin, neurofilament, synemins, and internexin are type IV IFs; lamins are type V IFs; and lens-specific phakinin and filensin are type VI IFs. IFs are located in the cytoplasm as keratins, desmin, vimentin, GFAP, neurofilaments, internexin, synemin, peripherin, and nestin, which are components of the cytoskeleton, or in the nucleus as lamins, where they form the nucleoskeleton. Lamins, which are classified into A and B type lamins, share structural features with cytoplasmic IFs but constitute thinner filaments of about 3.5 nm and carry a nuclear localization signal.

IFs participate in the connection between the cell membrane and the nuclear interior with actin filaments, microtubules, and the linker of the nucleoskeleton and cytoskeleton (LINC) complex. IFs are responsible for the critical structural integrity of cells and tissues and regulate several important cellular processes, including cell migration and adhesion, apoptosis, proliferation, differentiation, autophagy, signaling, gene expression, vesicle trafficking, mitochondrial function, and cell fate determination. Mutations in IF genes result in a wide range of human diseases; these filaments play important roles in the context of cancer, inflammation and other immune diseases, muscular syndromes, progeria, lipodystrophies, and digestive diseases. Although IFs are expressed in most cells, their expression is dynamic and subject to fine regulation involving post-translational modifications and intracellular proteolysis. In this Special Issue, the effects of deimination, a post-translational modification catalyzed by a calcium-dependent enzyme family of five peptidylarginine deiminases (PADs) were reviewed [1]. Deimination is related to physiological processes such as cell differentiation, embryogenesis, and innate and adaptive immunity, among others, and this modification has been associated with autoimmune diseases (rheumatoid arthritis, multiple sclerosis and lupus), cancers and neurodegenerative diseases. Deimination affects the polymerization and solubility properties of IF proteins and the proteolysis and cross-linking of IFs and IF-associated proteins.

Maggi et al. reviewed the recent discoveries related to skeletal and cardiac muscle disorders caused by mutations in the genes DES, SYNM, and LMNA encoding for the IFs desmin, synemin, and A-type lamins, respectively [2]. Concretely, they summarized clinical and molecular features of desmin, lamin, and synemin IF-associated striated muscle disorders and suggested pathogenetic hypotheses based on the interplay of desmin and lamin A/C proteins.

The complexity of the ovine and caprine keratin-associated proteins (KAPs) and their genes were reviewed [3]. The KAPs are structural components of wool and hair fibers used in clothing and furnishings, where they form a matrix that cross-links with the keratin intermediate filaments. This review consolidates the actual knowledge in identifying the KAPs and their genes in mammals and in determining how these genes and proteins are involved in fiber growth and characteristics.

Several studies describe major aspects of keratin basic physiology, their role in human diseases, and their potential regulation in therapy. One study demonstrated the interconnection and attachment of keratin IFs to the extracellular matrix via focal adhesions and hemidesmosomes. This manuscript also highlighted the fundamental role of hemidesmosomal adhesions for keratin network formation and organization independent of other cytoskeletal filaments [4]. Another study dissected the keratin-18-dependent expression of keratin 7 in the pancreas and the keratin 7 upregulation during experimental diabetes in mice [5]. Withaferin-A, an established disruptor of vimentin filaments, can also modulate keratin filament assembly in the absence of vimentin, as occurs in epidermal keratinocytes. This keratin disassembly is accompanied by variations in cell stiffness and migration, suggesting that Withaferin-A can be a valuable drug to disturb the keratin cytoskeleton against conditions that present excessive keratin assembly, such as hyperkeratosis [6].

Vimentin is a type III IF that is present in fibroblasts, endothelial cells, and cells of the immune system. Vimentin filaments form a network that extends from the nuclear periphery towards the plasma membrane of cells. Vimentin also exits as an extracellular protein in two forms: attached to the outer cell surface and secreted into the extracellular space. Vimentin has been involved in modulating the cell architecture, cell dynamics, migration, contraction, and endocytic and intracellular vesicle transport, among other cellular functions.

Several manuscripts described new aspects of vimentin. One of these demonstrated that Zinc, the transition metal that most frequently acts as a cofactor of proteins, reversibly induces the formation of vimentin oligomeric species. Thus, Zinc modulates vimentin organization, a fact that could have implications in physiological and pathophysiological processes, such as the human diseases acrodermatitis enteropathica and age-related macular degeneration [7].

Increasing studies have confirmed the interaction between caveolae and cytoskeleton, mainly with actin and microtubule networks, since the crosstalk between IFs and caveolae has remained elusive. One manuscript included in this Special Issue increased the knowledge regarding this still open field by describing that vimentin physically repressed the motility of cytoplasmic Cav-1 vesicles and inhibited the phosphorylation level of Cav-1, thereby modulating the role of Cav-1 on migration and wound healing. On the contrary, these authors showed that Cav-1 did not have a significant effect on the transcriptional, translational, post-translational modification, subcellular expression, and dynamics of vimentin [8].

Two studies addressed the role of vimentin or vimentin-interacting proteins in cancer cells. One of them used stimulated emission depletion (STED) super-resolution microscopy, live-cell imaging, and quantitative proteomics. This manuscript revealed a Histone Deacetylase 6 (HDAC6)-dependent increase in migration speed and loss of directionality connected to the main variations in the vimentin interactome in oncogene-expressing and metastasizing fibroblasts [9]. The other investigated the functional role of extracellular vimentin in non-tumorigenic and cancer cells through the evaluation of its effects on cell migration, proliferation, adhesion, and monolayer permeability. They showed that binding extracellular recombinant vimentin to the cell surface enhanced the permeability of both non-tumorigenic and cancer monolayer cells [10]. It has been shown that the receptor-binding domain of severe acute respiratory syndrome-related coronavirus-2 (SARS-CoV-2) spike protein can alter blood–brain barrier integrity, and the surface vimentin also acts as a co-receptor between the SARS-CoV-2 spike protein and the cell-surface angiotensin-converting enzyme 2 receptors. This study also investigated the influence of extracellular vimentin in SARS-CoV-2 infections. They observed that the enhancement of permeability from binding the extracellular vimentin to the cell surface is lost with cancer monolayers but not with non-tumorigenic cells upon the addition of the SARS-CoV-2 receptor-binding domain. This effect occurs because the extracellular vimentin binds directly to the viral domain, demonstrating extracellular vimentin’s influence on SARS-CoV-2 infections [10].

As previously mentioned, extracellular vimentin can act as a receptor for bacterial and viral pathogens playing important roles in virus attachment and entry not only in SARS-CoV but also in dengue and encephalitis viruses, among others. This topic was reviewed in this Special Issue by Ramos et al. [11]. In this manuscript, they recapitulated some of the pathophysiological implications of vimentin, including the involvement of cell surface vimentin in interaction with pathogens, with a special focus on its role as a cellular receptor or co-receptor for viruses. In addition, the authors provided a perspective on approaches to target vimentin, which could be useful when multi-target antiviral strategies are needed. These approaches included antibodies or chemical agents that could modulate the vimentin–pathogens interaction to potentially interfere with viral pathogenesis.

The nuclear envelope A- and B-type lamins are type V IF proteins. Mutations in the genes encoding these lamins cause rare diseases, collectively called laminopathies. Moreover, increasing experiments are describing the role of these lamins in the immune system. In this Special Issue, two manuscripts show recent advances on these two topics. Regarding laminopathies, one of them examined microscopy methods to compare normal cells and cells from laminopathy patients [12]. In this study, they showed the suitability of an immunofluorescence staining approach and super-resolution imaging by STED microscopy to study the lamin A and B network. These authors also demonstrated structural lamina differences between normal and laminopathy patient fibroblasts (LMNA c.1130G>T (p.(Arg377Leu)) variant) employing these approaches. Regarding the role of lamin A/C in the immune system, the second manuscript discussed and summarized studies that have addressed the role played by lamin A/C in the functions of innate and adaptive immune cells in the context of human inflammatory and autoimmune diseases, pathogen infections, and cancer [13].

Some other studies in this Special Issue described novel advances in the knowledge of other IFs or IF-related proteins. One of them identified a novel link between the IF organizer IFO-1 and cholesterol metabolism in the Caenorhabditis elegans intestine. The author showed that the disruption of the apical IF network disturbs the uptake of cholesterol, thus resulting in the dysregulation of cholesterol-dependent cellular processes and physiology [14]. In this group of manuscripts, one study showed that the IF desmin could directly bind to mitochondria; therefore, disturbing the desmin network may provoke an alteration in mitochondrial distribution, morphology, and functions [15]. Regarding the IF type IV synemin in the context of radioresistance and DNA repair in tumors, Deville et al. demonstrated that synemin modulated c-Abl phosphorylation and activity and governed a fundamental role in DNA double-strand break repair by serving as a kinase anchoring protein for an ataxia-telangiectasia mutated (ATM)-dependent interaction with c-Abl in head and neck squamous cell carcinoma [16].

Epithelial-to-mesenchymal transition (EMT) is a malignant cancer phenotype characterized by augmented invasion and metastasis. In this process, epithelial cells loosen cell–cell adhesion structures, including adherent junctions and desmosomes, modify their polarity, and reorganize their cytoskeleton. IFs usually change from cytokeratins to vimentin. The heparin-binding protein Fibroblast growth factor 2 (FGF2) is a common EMT-inducer of epithelial cells in vitro. In one manuscript of this Special Issue, the generation of engineered FGF2 dimers that mimic the natural FGF2 dimerization, a process that is essential for the formation of active ligand-receptor complexes, is described. Engineered FGF2 dimers showed increased stability, mitogenic potential, and anti-apoptotic activity compared to FGF2 monomers. These engineered dimers also induced greater migration responses in normal fibroblasts compared to FGF2 monomers [17].

An analysis of articles published in this Special Issue, entitled “Recent Advances in Intermediate Filaments”, showed new advances in important cellular functions regulated by IFs, such as proliferation, migration, cell stiffness, cell directionality, cell permeability, vesicle trafficking, mitochondrial function, wound healing, and cholesterol metabolism. These functions of IFs were relevant in different human diseases, such as skeletal and cardiac muscle disorders, diabetes, acrodermatitis enteropathica, age-related macular degeneration, cancer, pathogen infections, and laminopathies, highlighting the importance of IFs in different tissues and cell types.
